# The importance of parents’ communication and social environment in childhood cancer

**DOI:** 10.1007/s00431-025-06295-2

**Published:** 2025-07-25

**Authors:** Kleanthis Nizamis, Vassilios Kalliakmanis, Nikos Koutsoupias, Sophia Polychronopoulou, Margarita Baka, Evgenia Papakonstantinou, Emmanouil Hatzipantelis

**Affiliations:** 1 School of Theology, Aristotle University of Thessaloniki Greece, Thessaloniki, Greece; 2 Department of International and European Studies, University of Macedonia, Thessaloniki, Greece; 3 Department of Pediatric Hematology-Oncology, Aghia Sophia Children’s Hospital, Athens, Greece; 4 Department of Pediatric Oncology, General Children’s Hospital of Athens Panagiotis & Aglaia Kyriakou, Athens, Greece; 5 Pediatric Oncology Department, Hippokration General Hospital of Thessaloniki, Thessaloniki, Greece; 6 School of Medicine, Aristotle University of Thessaloniki, Thessaloniki, Greece

**Keywords:** Childhood cancer, Adolescents, Parents, Communication, Social environment, Quality of life, Serious illness

## Abstract

Parents facing child’s life-threatening diseases like cancer encounter a myriad of emotional challenges, often exacerbated by communication barriers. This study designed to explore the significance of effective communication and emotions’ management in improving the well-being and quality of life of these parents. The research was conducted on a sample of 133 families of children with cancer, and the results were displayed after statistical processing and data analysis with R statistical software. The results of the study confirm with statistically significant data, the importance of the communication and emotions’ management of the parents during the disease of their children, and the need of a multidisciplinary approach involving healthcare providers, psychologists, social workers, and support groups. Thus, 74.5% of the respondents stating that they receive help from their partners during the period of childhood cancer and 76.7% could also communicate and receive help from the other family members. The key findings are characterized by high specificity as it is a part of a unique study that reveals particular aspects of the Greek parent’s behavior, communication, and psychosocial problems during the period of their child’s illness.

## Introduction

Childhood cancer is an emotionally, physically, and financially devastating experience for families. It encompasses a broad spectrum of challenges that significantly impact every aspect of family life. These challenges are not limited to the immediate medical concerns but extend to emotional, psychological, and social domains [1]. Families grappling with a child’s cancer diagnosis encounter frequent treatments and hospitalizations, deal with secondary effects of the treatments, and live with ongoing uncertainty and fear of relapse [2, 3]. This uncertainty of the disease’s course add to the psychological strain, while social isolation and long-term consequences further complicate the family’s efforts to regain a sense of normalcy and a quality of life [4].

Communication plays a pivotal role in the healthcare journey of patients with life-threatening illnesses. Effective communication between healthcare providers, patients, and their families fosters trust, enhances understanding, and enables shared decision-making. This type of communication is a social support in the lives of parents whose children are battling cancer [5]. Emotional, informational, and practical support each address different aspects of the challenges these parents face, collectively contributing to their ability to cope and care for their child [6].

Communicating a diagnosis of a serious, potentially disabling disease is one of the most challenging aspects of health care [7]. However, by approaching this task with clear communication, and a commitment to supporting the patient’s emotional well-being, we can help them navigate this difficult journey. The way that we deliver these news can profoundly affect the patient’s ability to cope, adapt, and ultimately find hope in the situation. It is a challenging issue to focus on the vital aspect of managing reactions and emotions during this difficult.

The biggest challenge for the parent, beyond the set of diverse problems analyzed at length, is the management of their child’s illness in relation to the child himself. A frequent phenomenon that is observed is that parents are unable to manage their behavior, often losing their temper in contact and communication with their child. Many times children manage their health problem better themselves, with greater maturity compared to their parents. However, the lack of mental strength and the parents’ inability to manage the child’s disease is an important factor in deregulating the family balance, as the child perceives and experiences this weakness of his parents [3].

Healthcare providers must possess the empathy and communication skills necessary to navigate sensitive conversations, address patients’ emotional needs, and provide appropriate support throughout the treatment process [8, 9]. Implementing effective communication and emotions’ management strategies requires a multidisciplinary approach involving healthcare providers, psychologists, social workers, and support groups. The patient, as well as his family and social environment, have the need to come into contact with people who can bear his pain, without this meaning that they feel sorry for him. They also know how to manage and provide him with the space and time to exploit his mental and physical reserves, in order to maintain as much as possible an optimistic mood in the unpleasant climate created by the disease itself. In conclusion, effective communication and emotions’ management are indispensable components of comprehensive care for patients suffering from life-threatening diseases [10].

In this study, the answers that were collected from 133 families of children with cancer in Greece present a thorough analysis of how and to whom of their social environment the parents of a child with cancer communicate their physical, psychological, social, and practical problems.

## Materials and methods

In Greece, 300–350 cases of childhood cancer are reported every year. The study is a part of a PhD and a postdoc researches, which were conducted on a sample of 133 families of children with cancer and the results were displayed after statistical processing and data analysis with R statistical software. It is therefore easily understood that the research covered more than one-third of the total annual population of childhood cancer that exists in the country.

Four Pediatric Oncology Departments of Greek hospitals took part in this study. More specifically, we studied 64 cases from the Pediatric Oncology Department of the “Hippocration” General Hospital of Thessaloniki, 26 from the Children’s and Adolescent’s Hematology-Oncology Unit of 2nd Paediatric Clinic of A.U.Th. in the AHEPA General University Hospital of Thessaloniki, 25 from the Oncology Department of the “Panagiotis and Aglaia Kyriakou” Children’s Hospital, and 18 from the Department of Pediatric Hematology-Oncology of “Agia Sophia” Children’s Hospital.

The aim of the empirical research was to gather questionnaires within 1 full calendar year, in order to collect a sample based on the total cases of childhood cancer that we have in our country. Participation in the research was carried out through the completion of a closed questionnaire, which processed statistically in order to provide findings and results. For the compilation of the questionnaire, researches of other universities and research centers were taken into account on issues of management of childhood illness and specifically of neoplasia by parents and relatives.

In the main part of the questionnaire, a series of statements regarding the period of the child’s health issue are listed, in which each participant notes the extent to which each statement applies to him, expressing his agreement on a 7-point Likert scale. In addition, there are some questions in the form of multiple choices. The questionnaire consists of questions focusing on psychosocial and physical issues as well as communication and everyday issues. For example, there are questions like “during my child’s illness, when I needed help, I easily asked for it from my partner” or “during my child’s illness period, when I needed help, I received help from my family.” Statistical processing of the data was carried out by multivariate analysis, using methods of multiple correspondence analysis in a 0–1 table and automatic hierarchical classification. These methods were chosen as they allow the phenomenon to be examined as a whole, without assumptions and models [11, 12].

## Results

In the results of the study, we note that the parent shows relative comfort in requesting help and support from their partner during the child’s illness. Specifically, 49.6% scored the highest number 7 on the 7-point answer scale, a percentage to which if the answers of the two immediately following levels are added, it reaches to the 69.2% (Table 1). The numbers are also relatively high in seeking help from other members of the family, with a total of 61.6% in the three highest levels of responses expressing themselves positively in terms of the demand for support from family members (Table 1). Regarding the social environment, the numbers move to lower levels with only a cumulative 28.6% corresponding to the three highest positive responses (Table 1).

Moving now to the next question about getting help after asking for it, the numbers show a high response from partner and family, with a 51.9% highest positive response and a combined 74.5% in the top 3 positive responses regarding the partner and corresponding percentages of 50.4% and 76.7% regarding the rest of the family members (Table 2).

Regarding receiving help from friends and neighbors, the first group receives a cumulative 69.9% in the three highest levels of the 7-point Likert scale, with the second moving to particularly low levels with a cumulative 17.3% (Table 3).

Finally, in the results, we see answers about receiving help from the medical staff and parents of other children with cancer, where 67.6% express themselves positively, scoring the three highest levels regarding communication and support from the medical staff and 59.3% of parents noted positive answers when asked about their contact with other parents of sick children (Table 4).

## Discussion

During the intense period of childhood illness, some of the serious issues that arise are those of the expression of emotions, the parent’s need to communicate, and the management of his new reality. In previous related publications, we talked about the needs faced by parents of children with cancer in Greece, presenting a multitude of different issues, physical, psychological, and also spiritual, which each parent is called upon to resolve and also communicate about them by asking for help [13, 14].

The related literature presents high percentages of difficulties in communicating with the family circle, the social environment, medical and nursing staff, and a thorny continuity of daily previous activities [15–19], while others do not [20]. The results in the present research do not show high numbers. In particular, the participants do not have any particular difficulty in expressing their feelings towards their partner, family, and social environment during their child’s illness. On the contrary, we observe that they comfortably ask for help when they feel they need it, mainly from family and partner (Table 1). It is particularly worth pointing out that compared to a related study, which shows a percentage of 35% regarding receiving help from the family [17], in our research, the corresponding percentage is cumulatively for the three highest answers on the 7-point Likert scale, 76, 7%. (Table 2).

It is also worth mentioning that in addition to the family and partner that were previously analyzed, comfort of communication and high support and help based on the results are also received by the participants from other social groups. Specifically, cumulatively for the three highest responses on the 7-point Likert scale, friends gather 69.9%, medical/nursing staff 67.6%, and parents of other sick children 59.3% (Tables 3 and 4).

The literature argues for significant and beneficial outcomes that suggest the feasibility of encouraging functional coping strategies and social support in families of children with disabilities and chronic diseases. Cancer, congenital heart disease, diabetes, and autism are some indicative examples of diseases that related studies note the contribution of communication and social support of parents for a successful management of the disease within the family [21–26].

It is crucial to recognize and address the unique needs of parents during this tumultuous period and to help them navigate the complexities of the situation and communicate their problems effectively. The initial shock of the diagnosis is frequently followed by an overwhelming fear for their child’s life and future, compounded by the grief of disrupted dreams and expectations. This emotional upheaval can lead to significant stress and anxiety, potentially affecting their mental health and well-being. The anxiety created by the uncertain state of the child’s illness produced the need for communication and sharing of the difficulty [27]. High levels of depression and hopelessness state and trait anxiety scores for both mothers and fathers confirm the urgent need of psychological and social support to help parents cope with these problems [28].

Support for parents must encompass psychological counseling and emotional support. Access to professional counselors, support groups, and peer networks can provide a safe space for parents to express their fears and anxieties. Counseling can also equip parents with coping strategies to manage their emotions and maintain mental resilience. Support groups, where parents can connect with others facing similar challenges, offer a sense of community and shared understanding, reducing feelings of isolation and helplessness [29]. As we have seen in our study, in Greece, there are special emotional bonds between family and friends. We attribute the fact of the high positive responses according communication with all these support groups (Tables 1–4) to these strong emotional family and friendly ties that prevail in our country, with a strong willingness to share in the pain of each person.

Open and honest communication with healthcare providers and other support groups ensures that parents are fully informed about their child’s condition and treatment, fostering a collaborative approach to care. Clear communication within the family helps in maintaining emotional coherence and mutual support, crucial for the overall well-being of the child and family unit. In this point, we have to mention that the need for communication and support does not end with the conclusion of active treatment. The long-term effects of a chronic disability and especially childhood cancer, including the risk of recurrence and the emotional scars left by the experience, necessitate ongoing support for both the child and the parents [30, 31]. Survivorship programs and long-term counseling can provide continuous emotional and psychological support, helping families rebuild their lives and address any lingering fears or issues.

The above results testify that the immediate physical and functional problems of the child, with the repeated medical visits, the complex examinations, and the frequent hospitalizations, as well as the uncertainty about the future with the complex secondary psychological and social problems, cause stress to the child and his family, with the responsibility of treating the disease being shared between doctor, child, and family. The secondary effects of childhood illness, especially if it is a serious one such as neoplasms, constitute a threatening factor for a multitude of negative changes in the daily life of the parents that they have to communicate and share all this difficulty with someone who trust and can rely on.

## Data Availability

The datasets used and/or analysed during the current study available from the corresponding author on reasonable request.

## Appendix

**Table 1:** Asking for help during childhood cancer

During my child’s illness, when I needed help, I easily asked for it from:						
	Partner/husband/wife		Family		Social environment	
	Frequency	Percent	Frequency	Percent	Frequency	Percent
1	22	16.5%	21	15.8%	47	35.3%
2	7	5.3%	10	7.5%	18	13.5%
3	7	5.3%	11	8.3%	8	6.0%
4	5	3.8%	9	6.8%	22	16.5%
5	15	11.3%	14	10.5%	15	11.3%
6	11	8.3%	20	15.0%	10	7.5%
7	66	49.6%	48	36.1%	13	9.8%

**Table 2:** Receiving help during childhood cancer

During my child’s illness, when I needed help, I received it from:				
	Partner/husband/wife		Family	
	Frequency	Percent	Frequency	Percent
1	18	13.5%	8	6.0%
2	5	3.8%	5	3.8%
3	2	1.5%	7	5.3%
4	9	6.8%	11	8.3%
5	7	5.3%	16	12.0%
6	23	17.3%	19	14.3%
7	69	51.9%	67	50.4%

**Table 3:** Receiving help during childhood cancer

During my child’s illness, when I needed help, I received help from:				
	Friends		Neighbors	
	Frequency	Percent	Frequency	Percent
1	16	12.0%	80	60.2%
2	8	6.0%	13	9.8%
3	2	1.5%	8	6.0%
4	14	10.5%	9	6.8%
5	27	20.3%	8	6.0%
6	26	19.5%	7	5.3%
7	40	30.1%	8	6.0%

**Table 4:** Receiving help during childhood cancer

During my child’s illness, when I needed help, I received help from:				
	Medical staff		Parents of other sick children	
	Frequency	Percent	Frequency	Percent
1	21	15.8%	31	23.3%
2	4	3.0%	5	3.8%
3	4	3.0%	10	7.5%
4	14	10.5%	8	6.0%
5	26	19.5%	22	16.5%
6	21	15.8%	24	18.0%
7	43	32.3%	33	24.8%

1 = Strongly disagree.

2 = Disagree.

3 = Partially disagree.

4 = Neutral.

5 = Partially agree.

6 = Agree.

7 = Strongly agree.
